# Non-fatal disease burden for subtypes of depressive disorder: population-based epidemiological study

**DOI:** 10.1186/s12888-016-0843-4

**Published:** 2016-05-12

**Authors:** Karolien E.M. Biesheuvel-Leliefeld, Gemma D. Kok, Claudi L.H. Bockting, Ron de Graaf, Margreet ten Have, Henriette E. van der Horst, Anneke van Schaik, Harm W.J. van Marwijk, Filip Smit

**Affiliations:** Department of General Practice and Elderly Care Medicine, and EMGO+ Institute for Health and Care Research, VU University medical centre, Van der Boechorststraat 7, 1081 BT Amsterdam, The Netherlands; Department of Clinical and Experimental Psychology, University of Groningen, Groningen, The Netherlands; Netherlands Institute of Mental Health and Addiction, Utrecht, The Netherlands; Department of Psychiatry, and the EMGO+ Institute for Health and Care Research, VU University Medical Centre, Amsterdam, The Netherlands; Department of Clinical Psychology, and EMGO+ Institute for Health and Care Research, VU University and VU University medical centre, Amsterdam, The Netherlands; Department of Epidemiology and Biostatistics, EMGO+ Institute for Health and Care Research, VU University medical centre, Amsterdam, The Netherlands

**Keywords:** Depressive disorder, Subtypes, Burden of disease, Quality of life, Epidemiology

## Abstract

**Background:**

Major depression is the leading cause of non-fatal disease burden. Because major depression is not a homogeneous condition, this study estimated the non-fatal disease burden for mild, moderate and severe depression in both single episode and recurrent depression. All estimates were assessed from an individual and a population perspective and presented as unadjusted, raw estimates and as estimates adjusted for comorbidity.

**Methods:**

We used data from the first wave of the second Netherlands-Mental-Health-Survey-and-Incidence-Study (NEMESIS-2, *n* = 6646; *single episode* Diagnostic and Statistical Manual (DSM)*-IV depression, n* = 115; *recurrent depression, n* = 246). Disease burden from an individual perspective was assessed as ‘disability weight * time spent in depression’ for each person in the dataset. From a population perspective it was assessed as ‘disability weight * time spent in depression *number of people affected’. The presence of mental disorders was assessed with the Composite International Diagnostic Interview (CIDI) 3.0.

**Results:**

Single depressive episodes emerged as a key driver of disease burden from an individual perspective. From a population perspective, recurrent depressions emerged as a key driver. These findings remained unaltered after adjusting for comorbidity.

**Conclusions:**

The burden of disease differs between the subtype of depression and depends much on the choice of perspective. The distinction between an individual and a population perspective may help to avoid misunderstandings between policy makers and clinicians.

## Background

Depressive disorders affect 15 % of the population on a lifetime basis [1] and have a detrimental impact on social, family and professional role functioning [2–5]. Depression however, is not a homogeneous condition and its burden of disease might vary across Diagnostic and Statistical Manual (DSM)-IV subtypes.

Subtypes of depression can be classified into single episode or recurrent depression and then further graded by severity: mild, moderate or severe [6]. Burden of disease estimates for subtypes of depression have received remarkably little attention in research. Kruijshaar et al. [7] studied the associations of severity and type of depression with functional impairment of the individual in a Dutch general population sample. They concluded that recurrent depression was found not to be associated with more impairment than single episode depression. Higher severity classes however were associated with more impairment. In contrast, Vos et al. [8] arrived at the conclusion that recurrent depressions are associated with a greater burden of disease.

It should be noted that burden of disease can be assessed at the individual and at population level. At individual level, clinicians tend to give priority to the disorders that exact the heaviest toll on their patients while from a population perspective the disease burden might be driven by the number of people affected in addition to case severity and disease duration. Indeed, a study by Lokkerbol et al. [9] about the non-fatal burden of several mental disorders showed that the rank order of disorders by individual burden is often different from the rank order which is based on the population-level disease burden.

This current study aims to estimate the non-fatal burden of disease for subtypes of depression from both an individual and population perspective. Distinguishing both perspectives may clarify discussions about resource allocation. In addition, we take into account the impact of comorbidity. Estimating the disease burden with and without adjusting for comorbidity addresses two fundamentally different questions. When adjusting for comorbidity, one addresses an (academic) question about a disorder’s unique contribution to the disease burden overall. When also incorporating the additional disability weights of comorbid conditions one addresses a (pragmatic) question how much people suffer from a disorder while taking the realistic perspective that in real life people are not adjusted for comorbidity.

Taking these notions as starting points, we hypothesised that from an individual perspective, single and recurrent depressive episodes exact the same toll on individual patients (H1). However, when assessed from population perspective, we hypothesised that recurrent depression would emerge as health care priority due to the large number of people affected by recurrent depressions (H2). After all, some 45 % of the depressed people experience recurrences, usually cumulating to seven or eight depressive episodes over the course of their life [7] and spending as much as 21 % of their lifetime in a depressed condition [8]. We also hypothesised that the non-fatal burden of disease of depression follows the gradient of symptom severity (H3). Finally, due to comorbid conditions that may lend extra weight to the burden of disease, we hypothesised that after adjusting for comorbidity, the burden of disease for major depression is lower for each subtype of depression (H4). These hypotheses may not be as straightforward as might seem because it may the combination of severity of a depression, the duration of episodes (e.g. the duration of depressive episodes might be shorter in recurrent depression [10], the number of depressive episodes (first episode or recurrent episode) and comorbidity that drive the actual burden of disease. Therefore, an empirical investigation is required.

## Methods

### Sample

Data were collected from the first wave (2007–2009) of the second Netherlands Mental Health Survey and Incidence Study (NEMESIS-2, *n* = 6646). The methods used have been described elsewhere in detail [11, 12]. Briefly, a multistage, stratified random sampling procedure was applied. A random sample of 184 of the 443 existing municipalities was drawn. In these municipalities, a random sample of addresses of private households from postal registers was drawn. Based on the most recent birthday at first contact within the household, an individual aged 18–64 with sufficient fluency in the Dutch language was randomly selected for interview. The study was approved by a medical ethics committee and respondents provided written informed consent. Selected households received a letter from the Dutch Minister of Health, Welfare and Sport, in which the study was explained and recommended. Households were contacted by phone or visited in person [if no phone number was available] at least ten times during November 2007 to July 2009. The response rate was 65.1 %.

The sample was fairly representative of the general Dutch population, but males, younger people (especially in the 18–24 age bracket), people who had attained fewer years of education, and those not in paid employment were somewhat underrepresented. Therefore, post-stratification weights were used in all analyses [11, 12]. After weighting, the sample followed exactly the same multivariate distribution over age, gender, marital status and urbanization (stemming from a rural or urban setting) as the population according to Statistics Netherlands 2013 [13]. 361 Respondents were diagnosed with depression in the past 12 months according to the Composite International Diagnostic Interview (CIDI) 3.0 [14].

### Availability of data and materials

The data on which this manuscript is based are not publicly available. However, data from NEMESIS-2 are available upon request. The Dutch ministry of health financed the data and the agreement is that these data can be used freely under certain restrictions and always under supervision of the Principal Investigator (PI) of the study. Thus, some access restrictions do apply to the data. The PI of the study is co-author of this paper and can at all times be contacted to request data.

At any time, researchers can contact the PI of NEMESIS-2 and submit a research plan, describing its background, research questions, variables to be used in the analyses, and an outline of the analyses. If a request for data sharing is approved, a written agreement will be signed stating that the data will only be used for addressing the agreed research questions described and not for other purposes.

### Measures

The *presence of mental disorders* (major unipolar depression and comorbid mental disorders) was assessed with the CIDI 3.0 [14]. The CIDI is developed by the World Health Organization and is a psychiatric interview generating 12-month and lifetime prevalence rates of the DSM-IV mental disorders. The CIDI 3.0 was first produced in English and underwent a rigorous process of adaptation to obtain a conceptually and cross-culturally comparable Dutch version [15, 16]. Clinical calibration studies in various countries [17] found that the CIDI 3.0 assesses anxiety, mood and substance use disorders with generally good validity in comparison to blinded clinical reappraisal interviews. Studies on earlier CIDI versions concluded that the CIDI assesses common mental disorders with generally acceptable reliability and validity [18, 19]. Comorbid mental disorders included any anxiety disorder, any substance use disorder, dysthymia and attention deficit hyperactivity disorder (ADHD). Eating disorders was not diagnosed in NEMESIS-2. A diagnosis of bipolar disorder in the last 12 months by definition excludes the diagnosis of depression in the same period and was therefore not taken into consideration.

The CIDI diagnostic interview was used to retrospectively classify *type* of depression (single or recurrent) and assess the *duration* (in days), spent in depression in the last 12 months. Recurrent depression was declared if a participant had one or more depressive episodes prior to the episode in the past 12 months. To study comorbidity and its effects on the burden of disease, we used the CIDI without imposing the rules for the hierarchy among the disorders, meaning that if a person manifests with two disorders, we count this as two distinct disorders and not as a single disorder.

The *severity* of the depressive episode of the past year was assessed with the widely used and validated Sheehan Disability Scale (SDS) [20]. The SDS is a self-report measure of condition-specific disability and was incorporated in all diagnostic CIDI sections. It consists of four questions, each asking the respondent to rate, on a scale from 0 to 10, the extent to which a particular disorder ‘interfered with’ activities in one of four role domains (home, work, social, close relationships) during the month in the past year when the disorder was most severe. ‘Severe’ depression cases score in the range of 7–10 in at least two areas of role functioning. ‘Moderate’ depression cases are those who score 4–6 in any domain. Remaining cases are defined ‘mild’ [21]. Depression cases with unspecified severity (missings on all domains of the SDS, *n* = 22) were re-scored, using two questions of the CIDI about possible disruption of work, social contacts or personal relations (question D28) and/or in daily routine (question D28a). ‘Severe’ depression cases score 4 or 5 (D28) and 1 or 2 (D28a). ‘Moderate’ cases score 3 (D28) and 1or 2 (D28a) or 4 (D28) and 3 or 4 (D28a). Remaining cases are defined ‘mild’.

*Disability weights (DWs)* are weight factors that reflect the severity of the disease on a scale from 0 (perfect health) to 1 (equivalent to death). DWs were obtained from the Medical Outcome Study Short Form 6 Dimensions (SF-6D) using Brazier’s algorithm [22], a well-validated algorithm that was applicable to our data . The SF-6D is a much used and well-validated instrument to estimate Health Related Quality of Life valuations (HR QoL) derived from the Medical Outcomes Study (MOS-36) [23]. It is of note that the SF-6D can describe as many as 18,000 health states, i.e. all the permutations of the items (1) physical functioning, (2) role limitations, (3) social functioning, (4) pain, (5) mental health, and (6) vitality, each of which has five or six possible answers. Brazier and colleagues used a sub-sample of 249 health states to elicit valuations in a representative sample (*N* = 836) from the general public in the UK. During a personal interview each respondent was asked to value the selected health states, and valuation was carried out using the standard gamble method, which was originally developed by Von Neumann and Morgenstern [24]. In standard gamble, individuals are asked to make a hypothetical choice between the certainty of living in that particular health state versus engaging in a treatment entailing a chance of getting well at probability P and dying at probability 1-P. The idea is that people are more willing to accept a risky treatment that involves a higher risk of dying when their HR QoL is poor.

Brazier and colleagues used the health state valuations obtained in an econometric model to predict the values of all 18,000 health states that can be described by the SF-6D and to assess DWs.

*Covariates* included gender, age and level of education. Moreover, we adjusted DWs for the presence of comorbid mental disorders (assessed with the CIDI) and somatic illnesses, which might inflate DWs. These *somatic illnesses* were based on self-reports and consisted of a list 17 chronic general medical disorders being treated or monitored by a physician in the 12 months preceding baseline, such as asthma, COPD, osteoarthritis, heart disease, peptic ulcer and diabetes. Comparisons between self-reports of chronic physical disorders and medical records show moderate to good concordance [25–27].

### Metrics of non-fatal disease burden

#### Individual perspective: Quality adjusted life year (QALY) decrements

A common metric to describe an individual’s health-related quality of life is QALY. A QALY gain is the amount of time (T) spent in a health state, multiplied by a valuation of that health state. This valuation is called ‘utility’ (U), which is anchored between 0 (‘death’) and 1 (perfect health). Utilities can be rescaled such that a higher score signifies poorer health and are then called DWs. In this burden of disease study we base our calculations on the DWs. As such, the focus of this disease burden paper is at QALY *decrements* instead of QALY *gains*. To illustrate, living 5 years with a DW of 0.34 is equivalent to (5*0.34=) 1.7 QALY decrements.

#### Population perspective: Years Lived with Disability (YLD)

At population level we are not looking at one single individual spending time (T) in a certain health state weighted by a disability weight (DW), but at (N) individuals spending variable amounts of time, T, in that health state weighted by DW. This results in *YLDs*. The amount of time individuals collectively spend in a health condition can be described by the number of *person-years (PYRS)*. When we want to describe the disease burden of depressive disorder from a population perspective we compute PYR and multiply these by the DW associated with that health state: YLD = PYR * DW.

Henceforth, we reserve the terms “QALY decrement” for disease burden from individual perspective and “YLD” for disease burden from population perspective.

### Analysis

DWs were estimated for the various types and severities of depression (single, recurrent, single mild, single moderate, single severe, recurrent mild, recurrent moderate, recurrent severe) in order to estimate QALY decrements and YLDs. Here, we took two approaches: one without, and another with adjustments for comorbid mental disorders and somatic illnesses (12-month depression and 12-month comorbid mental disorders).

- *QALY decrements not adjusted for comorbidity* were computed as the average DW of all respondents in a certain subtype of depression multiplied by the average time spent in a depressed health state in the last year. While this approach may portray a realistic picture of the disease burden of a disorder, it can be criticised for overestimating the disease burden attributable to a disorder when there are comorbid conditions that lend extra weight to the DW.
- *QALY decrements adjusted for comorbidity* can be computed in various ways [28]. In this study we corrected for comorbidity by regressing DWs on all the depression subtypes, other mental disorders and somatic illnesses. The regression coefficients were then interpreted as the DW of one depression subtype adjusted for comorbid mental disorders and somatic illnesses. The intercept (constant) in the regression models could be interpreted as the DW attributable to unobserved factors affecting HR QoL such as minor illnesses, accidents and conditions that were not measured. The DW of a disorder is the base-rate DW (intercept, a) plus the adjusted DW corresponding to this disorder (regression coefficient, b), thus: DW = a + b. In this way, the adjusted DWs were computed for all subtypes of depression and multiplied by the average time spent in depressed state to estimate adjusted QALY decrements.
- *YLDs not adjusted for comorbidity* per subtype were computed by multiplying DWs by the number of PYRs. PYRs were calculated as the number of affected people (1-year prevalence) multiplied by the total time spent in depression in the last year. To facilitate interpretation, results were standardised per one million persons, thus expressed as YLDs/mln.
- *YLDs adjusted for comorbidity* were corrected for comorbidity by regressing DWs on all the subtypes, comorbid mental disorders and somatic illnesses.

The Brazier algorithm we used was executed in Excel (version 11.0 for Windows, 2003) and can be obtained from John Brazier at Sheffield University. All other analyses (estimation of DW, QALY, YLD, PYRS and standard errors) were conducted in Stata (version 10.1 for Windows). As data were weighted, Stata’s procedure for design-based analysis and robust statistical techniques based on first-order Taylor-series linearization method were used to obtain correct sample errors.

## Results

### Sample characteristics

Demographic characteristics of the total NEMESIS-2 sample and of the 361 individuals with depression are shown in Table 1 (weighted). The depressed population includes more women, is less highly educated than the total population and suffers significantly more from comorbid disorders.

**Table 1:** Characteristics of the total NEMESIS-2 population (*n* = 6646) and respondents diagnosed with depression (last year-prevalence, *n* = 361), weighted^a^

	Total population Nemesis-2	Depressed population Nemesis-2	SE
n (percentage)	6646 (100 %)	361 (5,2 %)	–
Mean age (years)	41.6	40.2	1.038
Females (%)	49.6	60.2	0.035
Educational level high-cat 4^b^ (%)	28.0	21.45	0.025
Any 12-month anxiety disorder (%)	10.1	40.0	0.031
Any 12-month substance use disorder (%)	5.6	10.5	0.019
Any 12-month somatic illness (%)	31.6	46.9	0.040
Any 12-month dysthymia (%)	0.89	15.4	0.026
Any 12-month ADHD (%)	1.2	2.7	0.010
Mean duration episode last 12 months (days)	n/a	120.86	7.15
Single vs recurrent episodes (%)	n/a	32.7/67.3	0.038/0.040
Mild vs moderate vs severe (%)	n/a	7.6/36.1/56.3	0.015/0.032/0.033

*Abbreviations: ADHD* Attention Deficit Hyperactivity Disorder, *SE* standard error, *vs* versus

^a^Weighting based on city of residence, part of country (north, south, east, west) and on a specific weight-factor to correct for differences in the response rates in several socio-demographic groups and in the probability of selection of respondents within households

^b^Higher professional or university education

### QALY decrements not adjusted for comorbidity

**Fig. 1:**
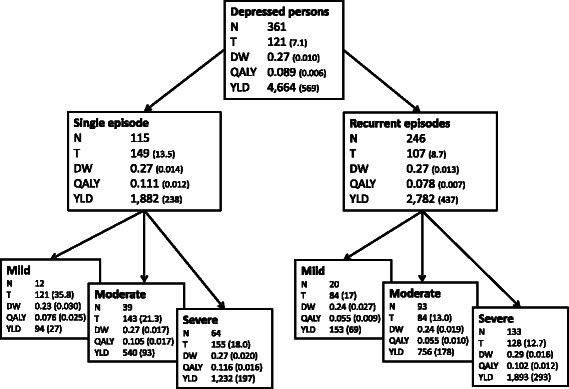
Unadjusted characteristics of burden of disease for subtypes of depression. Number of affected respondents (n), mean time spent in depression in days (T), disability weight (DW), quality of life decrement (QALY), years lived with disability per 1 million population (YLD) and standard errors (between parentheses) for major depression and subtypes of major depression; unadjusted for comorbid mental disorders and somatic illnesses. Standard errors of YLD were calculated using the standard rules when multiplying two variables, under the assumption that both variables (DW and PYRS/mln) are independent (se YLD/mln = √ ((se DW/DW^2^) + (se (PYRS/mln)/(PYRS/mln)^2^))

Figure 1 presents the number of affected patients, average duration of time spent in depression, DWs that were not adjusted for comorbidity, individual QALY decrements and YLD per one million for each of the subtypes in the population in the last year. The whole depression sample (*n* = 361) has, on average, a DW of 0.27 (se 0.010), meaning that people who meet the criteria for depression have a HR QoL that is 27 % lower than the non-depressed population. Single episodes have a longer duration than recurrent depressions (T = 149 and T = 107 days respectively). Regarding QALY decrements, a single episode poses a greater burden on individuals than a recurrent depressive episode (QALY = 0.111 and 0.078 respectively). Besides, higher levels of symptom severity are associated with higher QALY decrements.

### QALY decrements adjusted for comorbidity

**Fig. 2:**
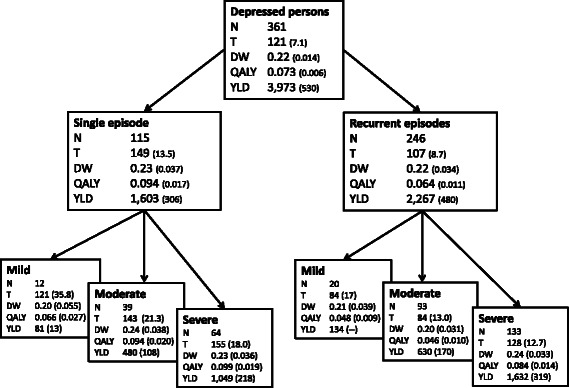
Adjusted characteristics of burden of disease for subtypes of depression. Overview of number of affected respondents (n), mean time spent in depression in days (T), disability weight (DW), quality of life decrement (QALY), years lived with disability per 1 million population (YLD) and standard errors (between parentheses) for major depression and subtypes of major depression; adjusted for comorbid mental disorders and somatic illnesses. Standard errors of YLD were calculated using the standard rules when multiplying two variables, under the assumption that both variables (DW and PYRS/mln) are independent (se YLD/mln = √ ((se DW/DW^2^) + (se (PYRS/mln)/(PYRS/mln)^2^))

Figure 2 presents the results when adjustments are made for comorbidity. Both unadjusted and adjusted QALY decrements show more or less the same ranking of results; single depression emerges as the leading causes of non-fatal disease burden (QALY =0.111). It appears that the adjusted QALYs are lower than the unadjusted QALYs by 18 % on average. The QALYs for single depression became smaller after adjusting for comorbidity than those for recurrent depression (0.017 versus 0.014 respectively).

### YLDs/mln not adjusted for comorbidity

From population perspective, the number of affected people becomes an important driver of disease burden. It appears that from a public health perspective recurrent depression causes a larger disease burden than single episode depression (YLD/mln = 2782 and 1882 respectively). Again, higher levels of symptom severity are associated with a greater burden of disease. Severe single episode and recurrent depressions emerge as the subtypes associated with the highest levels of YLD disease burden. Both types of depression are associated with a relatively large number of person-years and in addition have a high average DW, making them both leading causes of disease burden as seen from a population perspective.

### YLDs/mln adjusted for comorbidity

From population perspective, YLDs/mln are on average 15 % lower after adjustment. Comparable to disease burden at individual level, both the unadjusted and adjusted YLDs show a similar hierarchy: recurrent depression emerges as the leading cause of non-fatal disease burden (YLD/mln = 2.267). Single mild depression is the least disabling condition, mainly due to the small number of affected people.

## Discussion

### Main findings

This study estimated the non-fatal burden of disease for subtypes of depression such as single episode and recurrent depression, graded by severity (mild, moderate, severe depression). All estimates were assessed from an individual or a population perspective. In addition, the disease burden estimates were adjusted for comorbidity.

We hypothesised that from an individual perspective, the disease burden of a single depressive episode would be equal to the disease burden of a recurrent depression. However, results show that a *single* episode is associated with greater non-fatal disease burden than recurrent depression. Apparently, DWs are equal (both 0.27) but the time spent in recurrent depression versus a single episode is shorter (107 vs 149 days respectively).

The other three hypotheses were confirmed; from a public health perspective, *recurrent* episodes are associated with greater disease burden (H2). Even though the mean time spent in a recurrent depression is shorter, the large number of people affected comes into play (*n* = 115 single episode and *n* = 246 recurrent episode). As expected, the burden of disease follows the gradient of symptom severity (H3). Finally, the burden of disease for each subtype of depression is lower after adjusting for comorbidity (H4).

### Context and other studies

We need to place our findings in the wider context of the literature. Depressive disorder has been consistently identified as a leading cause of disability [29, 30]. Currently, depression is the single leading cause of non-fatal YLD disease burden in high-income countries and it is projected to become the second leading cause of DALY disease burden (which also accounts for mortality) by 2020, second only to ischemic heart disease [31]. More recent projections predict that depression might become the single leading cause of DALY disease burden in the high-income countries by the year 2030 [29]. The Global Burden of Disease (GBD) studies [32] conducted in 1990 and 2000 have quantified non-fatal health outcomes across a range of disorders at the global and regional level. The leading causes of YLDs were much the same in 2010 as they were in 1990, with depressive disorder contributing 8.1 % of total YLDs, ranking second after low back pain. Depressive disorder caused 63 million YLDs globally, but this figure was not disaggregated across the various types of depressive disorder. More recently, another GBD publication [4] showed that depressive disorders accounted for most YLDs within the group of mental and substance use disorders (42.5 %, 95%CI: 33.3–51.7)).

Kruijshaar et al. [7] studied the associations of severity and type of depression with functional impairment of the individual in a Dutch general population sample. Functional impairment was defined as limitations in physical, psychological and social functioning. Impairment was measured using the Short-Form-36 Health Survey (SF-36) [33] and two other indicators of impairment were added to reflect some of the economic consequences of depression as well: the number of days spent in bed due to psychiatric, drug- or alcohol-related problems, and the time missed from work due to these problems. The study did not make a difference between disability at individual or population level. They concluded that higher severity classes were associated with more impairment. However, recurrent depression was found not to be associated with more impairment than single episode depression. In contrast, Vos et al. [8] arrived at another conclusion. Their study focused on quantifying the burden of disease currently averted in people seeking care for major depression and the amount of disease burden that could be averted in these people under optimal episodic and maintenance treatment strategies. Results suggested that recurrent depression is the key driver of disease burden and that depression should be treated as a chronic episodic disorder in order to reduce this great burden of disease.

A study by Lokkerbol et al. [9], aimed at estimating the non-fatal burden of disease at both individual and population level due to several mental disorders in a Dutch population sample, estimated an unadjusted DW of 0.35 for depression and an adjusted DW of 0.25. YLD/mln were estimated 9117 and 6524 respectively. These findings showed that comorbidity plays an important role in causing disability, analogue to our findings. Contrary to our findings however, Lokkerbol’s adjusted and unadjusted DWs and YLD/mln led to different rank orders. However, Lokkerbol et al. used the first NEMESIS data [34] and as a consequence, their analyses were based on DSM-III-R disorders while ours were based on DSM-IV disorders. The fact that, from an individual perspective, the burden of disease for single depression decreased more than the burden of disease for recurrent depression after adjusting for comorbidity (0.017 versus 0.014 respectively) indicates that single depression is more often comorbid with other conditions.

### Strengths and limitations

The strengths of this study include the population-based representative dataset on which the analyses are conducted and using valid instruments like the CIDI and the SDS.

Another strength is that the disability weights were derived from the general population. The most recent GBD study [35] also accommodated data collection through population-based household surveys rather than from expert panels. This is important, because there are several ways of eliciting these valuations (e.g. from professionals in the medical field), but these are surrounded by controversy, and ultimately we need to understand how people value their own health [36].

A third strength is that we could compute both unadjusted and adjusted estimates. Unadjusted QALY decrements and YLDs portray an accurate picture of the burden of disease in subtypes of people that are likely to have comorbid conditions—after all, in real life, we do not encounter people who have been adjusted for comorbidity. Therefore, unadjusted estimates may have value from a public health perspective. However, when the aim is to assess the burden of disease attributable to a specific type of disorder, then adjusted estimates are preferred, because adjusted estimates give information about the level of disability due to a single disorder. In our study, we explored both approaches and were thus able to shed light on the different conclusions that depend on the chosen perspective.

A final strength is the distinction between a clinical perspective on individuals and a public health perspective on populations. This distinction might be important to inform clinicians, decision makers and researchers in the health care sector in a conceptually clear and unambiguous way.

We acknowledge the following limitations of our study.

First, we used a 1-year timeframe for the analysis of disease burden. Therefore we might have missed the intermediate dynamics of incidence, remission and recurrence within that year. Perhaps more importantly, the longer-term dynamics of the epidemiology of depressive disorder beyond 1 year were missed in our study. To illustrate, Vos and colleagues [8] estimated that in people with a history of depression the risk of a recurrence is 60 % within the 1st year after remission of the index episode, 70 % after 2 years, 80 % after 20 years and might be as high as 90 % on a lifetime basis. Despite this limitation, our approach is well-accepted and is used in the most recent WHO Global Burden of Disease (GBD) study [5], which aims to estimate the burden of disease consistently across diseases, risk factors and regions. To estimate burden of disease the GBD study was based on point (current/past month) or past year prevalence estimates and excluded lifetime estimates as recall bias invalidated them as credible measures of disease burden.

Second, we focused on the non-fatal disease burden, ignoring excess mortality, but elsewhere we computed that the risk of premature death is a factor 1.65 higher in people with depression as compared to people without depression [37]. Especially when taking the life-course perspective, mortality may have significant impacts on disease burden, and these impacts were missed due to our focus on ‘instantaneous’ non-fatal disease burden.

Third, people with severe conditions may have been unable to participate in this population-based survey, because they were hospitalised, and this is likely to have resulted in an under-estimation of the disease burden in the more severe forms of depression.

Fourth, we used the Brazier algorithm to calculate utilities and disability weights, but this algorithm was based on health state valuations in a sample of British people, while our sample was Dutch. This may have distorted our outcomes somewhat, although it is unlikely to change the overall results in a material way, as differences in Western Europe between national value sets, such as the set for the SF-6D, are small [38]. While we feel that the people should be the ultimate judges of their own health, a panel of laypeople may be associated with limitations that are worth noting, such as lesser consistency, and the possibility that (healthy) laypeople have difficulties passing judgments on the severe conditions. This may have caused some errors in the estimation of the disability weights associated with the more severe disorders. In fact, regarding the more severe conditions, Brazier et al. [22] pointed out that ‘inconsistent estimates (…) of the value of the poorest health states’ might be seen as a limitation of their method.

A fifth limitation is based on the fact that we relied on the DSM-IV diagnosis of major depression thus to the exclusion of minor depression which only became recognised as a disorder in the DSM-5. Nevertheless, it should be noted that minor depression also causes disability and is more common [39]. This findings may have resulted in an under-estimation of population estimates of disease burden, which depends on the proportion of people affected.

The sixth limitation is concerned with the validity of asking someone who may be depressed about their quality of life. Their answers may be biased because of the negative perceptive biases so common in depression. Therefore, we expect a downward bias in the estimated QALYs of depressed people and conversely an upward bias in the estimation of YLD.

A seventh limitation is about the standard gamble technique. It is a well-tested technique and is consistent with QALY theory [40]. However, in practice, appropriate adjustments for time preferences (i.e. discounting) for trade-offs made over a long period of time may be challenging to apply [41]. This technique may result in a lack of sensitivity if the respondent does not perceive the temporary health state as sufficiently impaired to induce them to trade time from their life or to gamble with a probability of death. This ‘ceiling effect’ may occur despite the presence of disutility from the impaired health state [42].

An eighth limitation is that data on somatic illnesses is based on self-report rather than diagnostic assessment, as well as the fact that we did not adjust for every illness, but only for 17 illnesses. This may have inflated DWs and YLDs estimates for depressive disorder.

A ninth limitation is that we did not adjust for *severity* of comorbidity, which might have provided a more realistic picture.

A final point: our analyses were performed on a sample that was diagnosed with depression during the past year, whereas the MOS SF-36 is designed to assess disability over the period of the past 4 weeks. The depressive episode may have occurred well before the last month as the average duration of an episode is 4–6 months. As a result, past month level of disability may not be truly representative for the disability that was actually experienced during the disorder. The individuals that are no longer suffering from depression may have therefore contributed to an underestimation of DWs, QALY decrements and YLDs. The use of a 12-month timeframe to estimate 1-month disability has the advantage that remitted disorders which continue to have residual adverse effects on disability are included. In sum, we captured the average effect of both acute and remitted episodes in the past 12 months on disability.

In this paragraph we mentioned a number of limitations. If these limitations led to bias overall, this bias will probably have led to an underestimation of the burden of disease but will probably not (or at least to a lesser degree) have impacted the ranking of the subtypes of depressive disorder.

### Implications

Major depression is a leading cause of non-fatal disease burden, but it is not a homogeneous condition and assessing the disease burden for subtypes of depression and according to a gradient of symptom severity may help prioritise treatment allocation.

Overall, we saw that the disease burden differs from one subtype to another and that comorbidity influences the results. In addition, our study showed that the burden of depression poses a substantial challenge both from a clinical perspective (at individual level) as well as seen from a public health perspective (at population level). This justifies the prevailing dichotomy of medicine into clinical medicine (directed at individual patients) and public health (directed at collectives). Health care providers who focus on helping individual patients may conclude from our results that a single episode depression causes a greater burden of disease than a recurrent depression. However, the high prevalence of recurrent depression in the population does raise questions about how to best alleviate the disease burden stemming from recurrent depression when taking the public health perspective. The distinction between a clinical and a public health perspective may cause confusion in debates when not made explicit.

Our message is that individual and population perspectives are neither absolute nor independent concepts. Both perspectives serve different purposes and may require careful alignment when being used jointly. Such an alignment may result in the optimal balance between an individual approach directed, for example, at the episodic treatment of acute single episode depressions, in combination with a public health care approach with an emphasis on the longer-term preventive management of recurrences.

## Conclusions

The burden of disease differs between subtypes of depression and depends much on the choice of perspective. The distinction between an individual and a population perspective may help to avoid misunderstandings between policy makers and clinicians.

## Ethics approval

NEMESIS-2 was conducted with the approval of the ethics committee of the Netherlands Institute of Mental Health and Addiction, Utrecht, the Netherlands and have therefore been performed in accordance with the ethical standards laid down in the 1964 Declaration of Helsinki and its later amendments. Respondents provided informed consent according to the prevailing Dutch law of 1996 after having been informed about the aims of the study.

## Abbreviations

ADHD: attention deficit hyperactivity disorder
CIDI: Composite International Diagnostic Interview
DSM: diagnostic and statistical manual (of mental disorders)
DW: disability weight
GBD: Global Burden of Disease
HR QoL: Health Related Quality of Life
MOS: Medical Outcomes Study
NEMESIS: Netherlands Mental Health Survey and Incidence Study
PI: Principal Investigator
PYRS: person years
QALY: Quality Adjusted Life Year
SDS: Sheehan Disability Scale
SF-36: short form 36 health survey
SF-6D: Medical Outcome Study Short Form 6 Dimensions
T: time
YL: years lived with disability

## References

[1] Bromet E, Andrade LH, Hwang I, Sampson NA, Alonso J, de Girolamo G (2011). Cross-national epidemiology of DSM-IV major depressive episode. BMC Med.

[2] Broadhead WE, Blazer DG, George LK, Tse CK (1990). Depression, disability days, and days lost from work in a prospective epidemiologic survey. JAMA.

[3] Kessler RC, Anthony JC, Blazer DG, Bromet E, Eaton WW, Kendler K (1997). The US national comorbidity survey: overview and future directions. Epidemiol Psichiatr Soc.

[4] Whiteford HA, Degenhardt L, Rehm J, Baxter AJ, Ferrari AJ, Erskine HE, et al. Global burden of disease attributable to mental and substance use disorders: findings from the Global Burden of Disease Study 2010. Lancet 2013;382(9904):1575–86. 10.1016/S0140-6736(13)61611-6 23993280

[5] Ferrari AJ, Charlson FJ, Norman RE, Patten SB, Freedman G, Murray CJ (2013). Burden of depressive disorders by country, sex, age, and year: findings from the global burden of disease study 2010. PLoS Med.

[6] World Health Organization (1992). The ICD-10 classification of mental and behavioural disorders: clinical description and diagnostic guidelines.

[7] Kruijshaar ME, Hoeymans N, Bijl RV, Spijker J, Essink-Bot ML (2003). Levels of disability in major depression: findings from the Netherlands mental health survey and incidence study (NEMESIS). J Affect Disord.

[8] Vos T, Haby MM, Barendregt JJ, Kruijshaar M, Corry J, Andrews G (2004). The burden of major depression avoidable by longer-term treatment strategies. Arch Gen Psychiatry.

[9] Lokkerbol J, Adema D, de GR, Ten HM, Cuijpers P, Beekman A, et al. Non-fatal burden of disease due to mental disorders in the Netherlands. Soc Psychiatry Psychiatr Epidemiol 2013 Feb 10. 10.1007/s00127-013-0660-8 23397319

[10] Spijker J, de GR, Bijl RV, Beekman AT, Ormel J, Nolen WA (2002). Duration of major depressive episodes in the general population: results from the Netherlands mental health survey and incidence study (NEMESIS). Br J Psychiatry.

[11] de Graaf R, Ten Have M, van Dorsselaer S (2010). The Netherlands mental health survey and incidence study-2 (NEMESIS-2): design and methods. Int J Methods Psychiatr Res.

[12] de Graaf R, Ten Have M, van Gool C, van Dorsselaer S (2012). Prevalence of mental disorders, and trends from 1996 to 2009. Results from NEMESIS-2. Tijdschr Psychiatr.

[13] CBS Statistics Netherlands (2013). Statistical yearbook of the Netherlands.

[14] Kessler RC, Ustun TB (2004). The world mental health (WMH) survey initiative version of the world health organization (WHO) composite international diagnostic interview (CIDI). Int J Methods Psychiatr Res.

[15] Alonso J, Angermeyer MC, Bernert S, Bruffaerts R, Brugha TS, Bryson H (2004). Use of mental health services in Europe: results from the European study of the epidemiology of mental disorders (ESEMeD) project. Acta Psychiatr Scand Suppl.

[16] de Graaf R, Ormel J, Ten Have M, Burger H, Buist-Bouwman MA, Kessler RC, Ustun TB (2008). Mental disorders and service use in the Netherlands. Results from the European study of the epidemiology of mental disorders (ESEMeD). The WHO world mental health surveys: global perspectives on the epidemiology of mental disorders.

[17] Haro JM, Arbabzadeh-Bouchez S, Brugha TS, de GG, Guyer ME, Jin R (2006). Concordance of the composite international diagnostic interview version 3.0 (CIDI 3.0) with standardized clinical assessments in the WHO world mental health surveys. Int J Methods Psychiatr Res.

[18] Andrews G, Peters L (1998). The psychometric properties of the composite international diagnostic interview. Soc Psychiatry Psychiatr Epidemiol.

[19] Wittchen HU (1994). Reliability and validity studies of the WHO—composite international diagnostic interview (CIDI): a critical review. J Psychiatr Res.

[20] Leon AC, Olfson M, Portera L, Farber L, Sheehan DV (1997). Assessing psychiatric impairment in primary care with the Sheehan disability scale. Int J Psychiatry Med.

[21] Ten Have M, Nuyen J, Beekman A (2013). de GR. Common mental disorder severity and its association with treatment contact and treatment intensity for mental health problems. Psychol Med.

[22] Brazier J, Roberts J, Deverill M (2002). The estimation of a preference-based measure of health from the SF-36. J Health Econ.

[23] Ware JE, The SCD, MOS (1992). 36-item short-form health survey (SF-36). I. Conceptual framework and item selection. Med Care.

[24] Von Neumann J, Morgenstern O (1953). Theory of games and economic behavior.

[25] Baker M, Stabile M, Deri C (2013). What do self-reported, objective measures of health measure?.

[26] Knight M, Stewart-Brown S, Fletcher L (2001). Estimating health needs: the impact of a checklist of conditions and quality of life measurement on health information derived from community surveys. J Public Health Med.

[27] National Center for Vital and Health Statistics (1994). Evaluation of national health interview survey diagnostic reporting. Series 2: data evaluation and methods.

[28] Andrews G, Sanderson K, Beard J (1998). Burden of disease. Methods of calculating disability from mental disorder. Br J Psychiatry.

[29] Mathers CD, Loncar D (2006). Projections of global mortality and burden of disease from 2002 to 2030. PLoS Med.

[30] Murray C, Lopez A (1996). The global burden of disease: a comprehensive assessment of mortality and disability from diseases, injuries, and risk factors in 1990 and projected to 2020.

[31] Murray CJ (2012). Disability-adjusted life years (DALYs) for 291 diseases and injuries in 21 regions, 1990–2010: a systematic analysis for the global burden of disease study 2010. Lancet.

[32] Vos T, Flaxman AD, Naghavi M, Lozano R, Michaud C, Ezzati M (2012). Years lived with disability (YLDs) for 1160 sequelae of 289 diseases and injuries 1990–2010: a systematic analysis for the global burden of disease study 2010. Lancet.

[33] Ware JE (1993). SF-36 health survey, manual and interpretation.

[34] Bijl RV, van ZG, Ravelli A, de RC, Langendoen Y (1998). The Netherlands mental health survey and incidence study (NEMESIS): objectives and design. Soc Psychiatry Psychiatr Epidemiol.

[35] Salomon JA, Vos T, Hogan DR, Gagnon M, Naghavi M, Mokdad A (2012). Common values in assessing health outcomes from disease and injury: disability weights measurement study for the global burden of disease study 2010. Lancet.

[36] Saarni SI, Suvisaari J, Sintonen H, Pirkola S, Koskinen S, Aromaa A (2007). Impact of psychiatric disorders on health-related quality of life: general population survey. Br J Psychiatry.

[37] Cuijpers P, Smit F (2002). Excess mortality in depression: a meta-analysis of community studies. J Affect Disord.

[38] Craig BM, Busschbach JJ, Salomon JA (2009). Keep it simple: ranking health states yields values similar to cardinal measurement approaches. J Clin Epidemiol.

[39] Kessler RC, Zhao S, Blazer DG, Swartz M (1997). Prevalence, correlates, and course of minor depression and major depression in the national comorbidity survey. J Affect Disord.

[40] Dolan P (2001). Output measures and valuation in health.

[41] Badia X, Monserrat S, Roset M, Herdman M (1999). Feasibility, validity and test-retest reliability of scaling methods for health states: the visual analogue scale and the time trade-off. Qual Life Res.

[42] Locadia M, Stalmeier PF, Oort FJ, Prins MH, Sprangers MA, Bossuyt PM (2004). A comparison of 3 valuation methods for temporary health states in patients treated with oral anticoagulants. Med Decis Making.

